# Lead Poisoning: An Alarming Public Health Problem in Bangladesh

**DOI:** 10.3390/ijerph6010084

**Published:** 2009-01-05

**Authors:** Amal K. Mitra, Akhlaque Haque, Manirul Islam, S.A.M. K. Bashar

**Affiliations:** 1 Department of Community Health Sciences, The University of Southern Mississippi, Hattiesburg, Mississippi, US; 2 Department of Government, University of Alabama at Birmingham; E-mail: ahaque@uab.edu; 3 North South University, Dhaka, Bangladesh; E-mails: manirul.raj@gmail.com (M. I.); skbashar@northsouth.edu (S. K. B.)

**Keywords:** Lead poisoning, children, Bangladesh, GIS, universal screening

## Abstract

To assess the risk of lead poisoning among preschool and school-aged children in Bangladesh, 345 children were screened for blood lead levels (BLLs) from one rural and two urban areas in Bangladesh from September 2007 through January 2008. An urban industrial area at Tongi was identified as a disaster area, where 99% (104/105) of those tested had BLLs ≥ 10 μg/dL. Industrial emissions and use of leaded gasoline by two-stroke engine vehicles were identified as possible sources of lead in that area. A rural nonindustrial area at Chirirbandar, Dinajpur was identified as another high-risk area, where 14% of the children screened had BLLs ≥ 10 μg/dL. BLLs at the urban industrial area were significantly higher than those at the rural and urban nonindustrial areas (24.58 ± 10.32, 7.24 ± 6.31, and 2.47 ± 3.32 μg/dL, respectively; p <0.001). Weight-for-age z-scores of the urban children were significantly lower than that of the rural children (–1.41 ± 1.88 vs. 0.20 ± 1.16, p <0.001). Children with elevated BLLs had poorer nutritional status (p = 0.05) than those with normal BLLs. Over 90% of the parents did not know that lead causes health problems. In conclusion, the problem of lead poisoning in children was found to be high in both urban and rural Bangladesh. A universal lead screening for preschool and school-aged children and a lead education program for parents are recommended for implementation in Bangladesh.

## 1. Introduction

Lead has long been recognized as a harmful environmental pollutant. In late 1991, the Secretary of the Department of Health and Human Services referred to lead as the number one environmental threat to the health of children in the United States [1]. According to the Centers for Disease Control and Prevention (CDC), an elevated blood lead level (BLL) is defined as ≥ 10 μg/dL [2]. In children, BLLs as low as 10 μg/dL, or even lower, have been associated with developmental delays, deficits in behavioral functioning [3, 4], decreased stature [5], diminished hearing acuity [6], and difficulty learning [7]. A 1 μg/dL increase in BLLs was associated with a 3.32 point decline in cognitive functioning in children ages six months to three years and a 2.47 point decline in children ages three to five years-old [8]. BLLs ≥ 70 μg/dL in children should be considered a medical emergency. This level of lead can cause serious health issues, such as seizures, coma, and death [9]. In the CDC’s 1991 revised document [2], universal screening of infants and young children in areas where lead exposure is still common was recommended.

In Bangladesh, an estimated 6.9 million children 5–14 years-old (12.9% of the total labor force) are engaged in physical labor. They are exposed to more than 200 hazardous and risky conditions, including welding, car repair, lead melting, ship breaking, and pottery glazing, all of which are likely sources of lead poisoning [10].

Air lead levels were recorded as being very high in Dhaka, Bangladesh between 1997–2000 [11]. In a cross-sectional study of 49 children from three areas in Dhaka, the mean ± SD of blood lead levels was 17.6 ± 4.9 μg/dL [12]. All children from the study areas exhibited elevated BLLs, possibly due to environmental exposure from gasoline, paints, ceramics, and used batteries. However, these study results are not representative of the Dhaka city population because the study was conducted only in high-risk populations. In another cross-sectional study of 779 school children in the city of Dhaka, nearly 90% had BLLs ≥ 10 μg/dL, 50% had BLLs ≥ 15 μg/dL, and almost 20% had BLLs ≥ 20 μg/dL [13].

Based on these limited data, lead poisoning is likely to be a major public health problem among Bangladeshi children. Some of the following factors may contribute to the problem. Although the government of Bangladesh adopted a policy to ban the sale of leaded gasoline in 1999, there is no complete control over the use of leaded gasoline in the country [14]. Law enforcement agencies do not monitor lead emissions via industrial waste disposal. The lack of a proper waste management system means that disposed batteries, pottery, and cans, (all good sources of lead), are available near areas where poorer children play. The lead content in pesticides and insecticides available in the market are not monitored. There is currently no routine screening or surveillance system in place in the country to monitor the level of children’s lead exposure. In addition, micronutrient malnutrition, which is common among Bangladeshi children [15], may predispose them to increased lead absorption and toxicity.

This study aimed to 1) assess the extent of problem of elevated BLLs among young children in both urban and rural Bangladesh; 2) identify children at risk of elevated BLLs in terms of socio-demographic and anthropometric parameters (weight and height); and, 3) locate cases with elevated BLLs and possible sources by using Geographic Information Systems (GIS).

## 2. Methods

The study was conducted from September 2007 through January 2008 in Bangladesh. A team of epidemiologists, two public health students, one laboratory technician, one GIS specialist, social workers from two non-governmental organizations, local political leaders, and school teachers participated in the selection of sites, lead education campaigns, and data collection for the project. The study was approved by the Human Subjects Protection Review Committee at The University of Southern Mississippi. Informed consent was obtained from parents of small children and assent was obtained from older children before enrollment.

**Figure 1.**
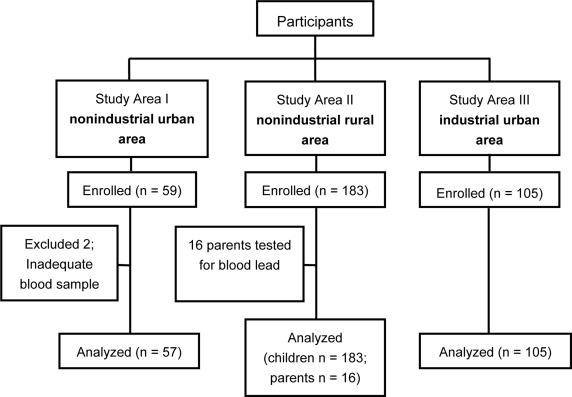
Sample areas and the study children in Bangladesh.

Samples of venous blood (0.5 ml) were collected from preschool and school children ages six months to 12 years from three locations: a periurban nonindustrial area at Azampur, Uttara (Study Area I); a rural nonindustrial area at Chirirbandar, Dinajpur (Study Area II); and, an urban industrial area at Rajabari, Turag, Tongi (Study Area III) (Figure 1). BLLs were measured using a portable LeadCare instrument (ESA Inc., Chelmsford, MA, USA). The instrument has been approved by the US Food and Drug Administration (FDA), and is being used in many health centers in the United States. The reliability and accuracy of the instrument was reported elsewhere [16]. To ensure data validity, the following measures were taken: (1) the principal investigator (AM) had received a hands-on training on using the portable LeadCare instrument; (2) blood samples from four volunteers were analyzed prior to using the instrument in the field level to ensure the laboratory procedures; (3) all blood samples were processed and analyzed by a single trained person (AM); (4) the instrument was calibrated each time before running a sample for analysis; (5) LeadCare blood lead controls were analyzed on each new lot of test kits used to monitor the accuracy and precision of the tests; (6) when a BLL was ≥ 30 μg/dL, the instrument was validated by calibration and a second blood sample was analyzed.

Children’s body weights were measured using a bathroom scale with an accuracy of 1 gm; while heights were measured using a standard mechanical stadiometer with an accuracy of 1 cm. Weight-forage z-scores were calculated using the National Center for Health Statistics standards.

Parents completed a pre-tested questionnaire (written in Bangla) regarding sociodemographic information, including child’s behavior, food habits, other habits, location and type of house the family lives in, the year the home was built, the home’s distance from a highway or intersection, and potential sources of lead exposure in the home environment. Once children were identified as having elevated BLLs, an in-depth household interview was conducted with parents to determine symptoms of lead contamination among the children. Children with BLLs ≥ 45 μg/dL were advised to take a chelating agent [17]. A chelating agent is a substance whose molecules form bonds to a metal, which aids in the metal being removed from the body. Parents were provided information regarding contamination and prevention methods, which followed the American Academy of Pediatrics recommendations [17].

### 2.1. GIS Mapping

Parameters such as location of homes, schools, industries, major roads, highways and intersections, and the location of person with elevated BLLs were geocoded, and GIS maps were constructed.

### 2.2. Statistical Methods

Data was analyzed using SPSS for Windows, version 15.0 (SPSS Inc., Chicago, IL, USA). BLLs of children were categorized into: <10 μg/dL; 10–14 μg/dL; 15–19 μg/dL; 20–44 μg/dL; 45–69 μg/dL; and ≥ 70 μg/dL. Mean BLLs were compared among the three study areas by one-way ANOVA and Tukey’s HSD tests. Children with normal and elevated BLLs were compared by Student’s *t*-test for continuous variables and by Chi-square test for categorical variables. A Mann Whitney Test was used for continuous variables with nonnormal distribution. A logistic regression analysis was conducted to identify which of the explanatory variables predicted elevated BLLs. Pearson’s correlation was used to assess correlation between BLLs and other variables. Distance between locations was measured by using the latitudes and longitudes of the points. A probability level of 0.05 or less was considered to be statistically significant.

## 3. Results

Of 347 children enrolled, two were excluded because of inadequate blood samples. Results presented in this paper represent data from 57 children in Study Area I, 183 children in Study Area II, and 105 children in Study Area III. Of these, 162 samples were from two urban areas near Dhaka (Study Areas I and III), and 183 samples were from a rural area at Chirirbandar, Dinajpur (Study Area II) (Figure 1). Of the total samples, 133 (39%) had BLLs ≥10 μg/dL.

### 3.1. Study Area I: Urban Nonindustrial Area (Azampur, Uttara)

**Figure 2.**
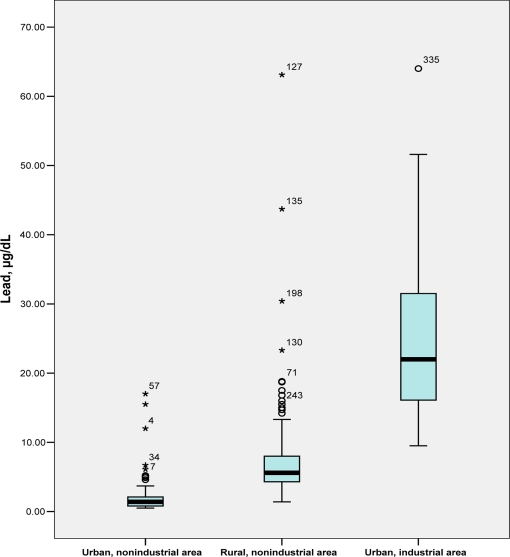
Blood lead levels of children in the three study areas. Numbers on the top of each bar in the box plot represent case identification numbers.

Azampur, Uttara is a periurban poor community located 15.9 km northeast of Dhaka. The average family income is Taka 3,500 ($45) per month. A typical family in this area consists of four or five people living in a small one room home, approximately 10–11 sq feet. Of the 57 samples collected from this area, three (5%) had elevated blood lead levels (Figure 2). The BLL levels ranged from 0.50 μg/dL – 17.0 μg/dL. Possible sources of lead in this area were small factories (welding and car repair workshops) located 1 km from the cases, a large dumping station for waste disposal located 1.1 km from the cases, and major intersections that were 1.7 km from the case locations.

### 3.2. Study Area-II: Rural Nonindustrial Area (Chirirbandar, Dinajpur)

Chirirbandar, Dinajpur is a small rural town, 270 km northwest of Dhaka, approximately 5.58 sq km in size. The population is 5,817, of which 54% are males and 46% are females. The population density of the area is 1,042 persons per sq km. At the time of the study only 38.8% of the population was literate, 37.1% were males and 19.5% were females. Most of the people in this area were employed in one of the following areas: agriculture (77.4%), wage labor (1.9%), weaving (1.8%), business (8.9%), and service (6.5%). The few industries in the village included brick kilns, fabric dyeing, and weaving factories.

Out of 183 samples, 26 (14%) had elevated blood lead levels, with a range of 1.4 μg/dL – 63.1μg/dL (Figure 2). In order to identify whether or not any adult family members (related to the children with elevated BLLs) also had elevated BLLs, 20 parents were approached and 16 consented to participate. Of the 16 parents, four (25%) had elevated BLLs. In one family (two parents and one child) each member tested had elevated BLLs. The sources of lead in this area were unknown. Welding and car repair workshops, located approximately 1.8 km from the cases, were a few suspected sources of lead poisoning.

### 3.3. Study Area-III: Urban Industrial Area (Rajabari, Turag, Tongi)

Of the three locations included in this study, the most devastating lead poisoning results were found in Rajabari, Turag, Tongi. This industrial area was located 18 km northeast of Dhaka. The major industries in the area were: battery, ceramic, pharmaceutical, cotton, jute, and plastics. The economic status of the people was average, with an income of Taka 8,000–10,000 ($125–$145) per month. Two major modes of lead transmission in this area were industrial discharges and the use of leaded gasoline in vehicles with two-stroke engines.

Out of 105 samples, 104 (99%) had elevated blood lead levels. The range of blood lead values was 9.5 μg/dL – 64.0 μg/dL (Figure 2). The average level of lead in Area III (24.58 ± 10.32 μg/dL) was significantly higher than Area II (7.24 ± 6.31), and Area I (2.47 ± 3.32) (p <0.001 for all).

### 3.4. Comparison Between Children With Elevated and Normal Blood Lead Levels

Table 1 shows that children with elevated BLLs were younger (p <0.001), and comparatively more malnourished (p = 0.05), compared to children with normal BLLs. A low weight-for-age z-score remained the significant predictor of elevated BLLs, after controlling for age in a logistic regression analysis.

### 3.5. Clinical Complaints of Selected Children

An in-depth interview of children with elevated BLLs and their parents showed that the majority of the children did not have any clinical symptoms, or had nonspecific symptoms. However, a number of parents complained about the stunted growth of their children. These children were also reluctant to play or participate in school activities (Table 2). One child suffered from seizures. A couple of children complained of fatigue, did not eat properly, and fell behind in school. One female child did not have any complaints and her weight-for-age z-score was normal.

### 3.5. GIS Mapping of Elevated Blood Lead Levels and Their Possible Sources

**Figure 3.**
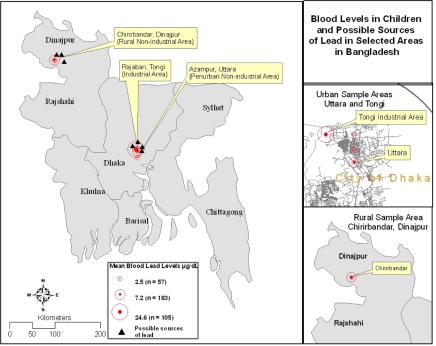
Location of sampling points for lead screening.

Figure 3 shows the locations of the study areas and the possible sources of lead. Since all cases were collected from one school in each area, and most of the cases were located very close to one another, they were not plotted individually.

**Figure 4.**
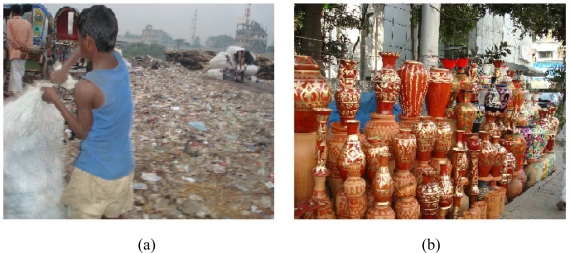
(a) A “Tokai” (poor street-boy) collects anything and everything from a garbage dumping place, and is likely to be exposed to environmental contaminants including lead; (b) The glazing material used for these flower-vases is a good source of lead.

As mentioned earlier, industrial discharges were the major possible source of lead poisoning in urban areas. Some other minor but possible sources of lead poisoning were the use of open fields for garbage disposal (Figure 4a) and pottery glazing (Figure 4b).

## 4. Discussion

The problem of lead poisoning in Bangladeshi children was high, with 39% of the study children having elevated BLLs. The industrial area at Tongi was identified as the worst affected area among the study population, having all but one sample with elevated BLLs. Industrial discharges and use of leaded gasoline in vehicles with two-stroke engines (locally known as baby taxis) were important sources of lead contamination in the population. Several industries including ceramic factories, battery production and processing factories, plastic manufacturing industries, chemical and pharmaceutical industries, cement production and processing factories, fertilizer factories, metal workshops, and car repair and welding workshops are good sources of lead in the major cities, especially at the Tongi industrial area, in Bangladesh. Although the National Environmental Council of Bangladesh banned the use of leaded gasoline in 1999, followed by the withdrawal of two-stroke engine auto rickshaws from the City of Dhaka in 2003 [14], leaded gasoline was still in use in vehicles outside the capital city. Studies in the United States have estimated that gasoline contributed to more than half of the blood lead burden in the 1970s when leaded gasoline was in use. Studies also suggested that a decrease of 100 metric tons of lead used in gasoline is associated with a decrease in mean BLL of 2.14 μg/dL [18].

Another finding of interest from this study was that the rural area of Chrirbandar, Dinajpur, where there were no major industries, was moderately affected, according to the percent of children with elevated BLLs. CDC guidelines define an area in the United States as being at high-risk if 12% or more of the children tested are found with BLLs ≥ 10 μg/dL [19]. There is no such cut-off point to define high-risk areas in Bangladesh. Based on CDC criteria, Chirirbandar, where 14% of the children had elevated BLLs, was considered a high-risk area. It should be emphasized that this study did not identify actual sources of lead. Further studies are needed to find out if major sources of lead poisoning are different in rural areas than urban areas. It is also important to establish a causal link between the suspected sources of lead and persons with elevated BLLs.

In many countries, including the United States, poverty is often associated with elevated BLLs, because children living in families whose incomes are below the poverty line have higher average BLLs than those living in families with incomes above the poverty line [20]. This study in Bangladesh was conducted among poor communities. Also, children with elevated BLLs were more malnourished when compared to those with normal BLLs. The direct relationship between elevated BLLs and poor nutritional status was also found in some other developing countries [21]. A diet rich in calcium and iron is necessary, because deficiencies in these minerals can increase the body’s absorption of lead, which explains why malnourished children are more prone to have high levels of lead in their blood [22].

In this study, children exhibited no specific clinical features that would identify them at risk of having elevated BLLs. That emphasizes importance of having a blood lead test done for the vulnerable population. However, an in-depth interview of parents revealed that children with elevated BLLs were less likely to eat properly and their growth was much slower. They were also reluctant to go to school and fell behind in academic performance. One child with a BLL level of 43.7μg/L had several seizures. However, the parents did not understand that this health issue was directly related to elevated BLLs in their child, nor did they think that lead poisoning is a big health problem. It should be noted that adverse outcomes, such as reduced intelligence quotient and academic deficits occur even with very low lead exposure [23]. No level of lead exposure appears to be safe and even the current recommended levels of lead <10 μg/dL in children are associated with neurodevelopmental deficits [23].

This study had several limitations. One of the limitations was that this study did not establish any causal pathway between suspected sources and cases of elevated BLLs. The nature of non-random sampling design has its potential weakness of bias. The samples were drawn from three areas, which may not sufficiently represent the total extent of the problem in the country. However, this study reinforced the severity of the problem of lead poisoning in young children in Bangladesh. Further studies are needed to identify the problem of lead poisoning in several other areas of Bangladesh and among people of different economic status. Based on this study, a universal lead screening program for preschool and school children in Bangladesh, as well as a mass lead education program to increase parents’ awareness of lead poisoning is recommended.

## Figures and Tables

**Table 1.** Characteristics between children with elevated blood lead levels and those with normal blood lead levels.

Characteristic	Children with blood lead levels		*χ^2^*	*p*-value
	< 10 μg/dL	≥10 μg/dL		
Age, mean ± SD	9.10 ± 3.83	7.74 ± 2.81	–	< 0.001^a^
≤6 years	58 (27.4%)	41 (30.8%)	27.85	< 0.001
7–12 years	115 (54.2%)	92 (69.2%)		
≥13 years	39 (18.4%)	0		
Weight-for age z scores	–.51 ± 1.86	–.63 ± 1.53	–	0.05^b^

^a^. Student *t*-test;

^b^. Mann Whitney test; blood lead levels of ≥ 10 μg/dL are considered elevated levels.

**Table 2.** Clinical complaints of selected children with elevated blood lead levels.

Case No.	Age (year) Sex	Blood μg/dL	lead	Weight-for-age z-score	Clinical complaints
1	12, M	38.2		–0.6015	Slow to answer very simple questions; has a blank look on his face. His mother says that he regularly complains about feeling weak and tired and sometimes gets headaches and feels nauseous. He does not feel like studying because it makes his head feel “heavy.”
2	10, F	21.0		–1.3338	She often feels tired and does not like studying.
3	9, F	43.7		0.2666	Sister of Case no. 2. She has a blank stare and recently had two attacks of seizures. She is taking medication, but was also given a tabij (amulet) to wear, which her mother believes will keep “evil spirits” away from her.
4	5, F	25.6		–1.4670	Besides occasional headaches and stomachaches she does not have any health problems.
5	12, M	15.5		–0.1940	Brother of Case no. 4. He hates studying and does not eat regularly. His mother says she has given up all hope on her son as he does not even have the patience to sit down and do his homework.
6	5.6, M	15.5		–2.7663	Weakness, cold, and fever.
7	2, M	12.0		–5.4723	Frequent attacks of diarrhea.
8	2, M	17.0		0.2366	No complaints.
